# Evaluation of Daylight Perception Assessment Methods

**DOI:** 10.3389/fpsyg.2022.805796

**Published:** 2022-04-11

**Authors:** Gizem Izmir Tunahan, Hector Altamirano, Jemima Unwin Teji, Cosmin Ticleanu

**Affiliations:** ^1^UCL Institute for Environmental Design and Engineering, The Bartlett, London, United Kingdom; ^2^BRE (Building Research Establishment), Hertfordshire, United Kingdom

**Keywords:** daylight availability, daylight perception, seat preference, drawing, environmental behaviour, method evaluation

## Abstract

Daylight is an important component in maintaining human health and wellbeing and plays a key role in physiological, psychological, and behavioural regulation. Understanding the complexity of daylight perception is vital since the degree of satisfaction with daylight conditions could greatly impact individual mood, behaviour and cognitive performance. This paper aims at (1) presenting an overview of current knowledge on methods for assessing daylight perception and (2) establishing a methodology for assessing daylight perception in the context of cultural background. An experiment was conducted with 50 students who were instructed to select the best and worst seats, describe the best desks’ daylight conditions and draw boundary lines between perceived daylit and non-daylit spaces in a library. The study showed that subjective rating and seat preference methods were consistent with actual daylight levels. However, participants’ boundary lines did not represent the actual daylight availability in the space. The study suggests that individual daylight perception in the context of cultural background can be assessed using the subjective rating and seat preference methods.

## Introduction

The characteristics of an indoor lighting environment could significantly affect the comfort, wellbeing and productivity of building occupants (Al horr et al., 2016). The lighting quality assessment typically includes photometric measurements, which does not provide a complete representation of an environment’s lighting quality (Allan et al., 2019). The assessment should not only consider the links between the lighting levels and the characteristics of the space where light is measured but, more importantly, how people perceive that environment. As of today, far too little attention has been paid to daylight perception and its evaluation methods, as highlighted by the Commission Internationale de l’Eclairage 2013 (CIE 213, 2014) and the Dubois et al. (2016). Understanding its complexity and potential benefits could be crucial, especially in the context of health and wellbeing, mood, and also cognitive and academic performance. Up to now, several studies have shown that exposure to different amounts and characteristics of daylight could enhance students’ cognitive performance (Shishegar and Boubekri, 2016; Jamrozik et al., 2019). However, it is still not known how students’ daylight perceptions and preferences and the level of daylight they are satisfied will contribute to their academic performance.

Culture, one of the essential components of an individual, delineates the characteristics of a group with similarities such as language, religion, tradition, and ethnicity. Knowing the cultural background of a group of people is vital because it could help understand why a group of people acts similarly compared to another group. Lighting research to date has tended to focus on the impact of cultural background on glare discomfort perception rather than daylight perception and satisfaction. Most cross-cultural lighting studies have examined discomfort glare perception and colour temperature preference, but they did not sufficiently focus on the adequacy of illuminance levels. Cross-cultural studies aiming to investigate lighting preferences in interior environments are rare and what is not yet known is the importance of cultural background and its impact on daylight perception, expectation and satisfaction.

In the field of lighting environment, Pierson et al. (2018) have used the term of ‘culture’ as ‘*the climatic and indoor conditions to which the subject has been accustomed during the major part of his/her life, his/her behaviour toward this indoor environment, and his/her expectations about it*’. Subsequently, a recent study (Izmir Tunahan et al., 2021) has highlighted the importance of cultural background in daylight perception and suggested that the cultural background in the lit environment should be evaluated, considering (1) the ethnicity and/or physiological characteristics of the individual eyes, (2) the area (luminance environment) where people used to live, (3) the luminance environment they were recently exposed to, and (4) the socio-cultural background of individuals.

In the United Kingdom, students constitute 19% of higher education (equals to 438,010 students) with 13.6% of undergraduate, 36.6% of postgraduate (taught) and 43.2% of postgraduate (research) students. They travel mostly from countries with a wide range of daylight conditions that differ from each other and from daylight conditions in the United Kingdom (e.g., China, Malaysia, the United States, Nigeria, India, Germany, France, Italy and Ireland; UKCISA, 2017). Outside daylight conditions refer to the amount and duration of daylight varying with the sun’s position in the sky depending on latitude and atmospheric conditions that depend on various factors (e.g., turbidity, climate and pollution). Hence, students from different parts of the world could be assumed to have previously experienced different lighting environments and students from locations with similar daylight conditions should have comparable daylight expectations. To this end, students’ cultural diversity and the specific lighting environments they were previously accustomed to could affect their perception and expectation towards the outdoor and indoor conditions they found in the United Kingdom (Izmir Tunahan et al., 2021).

Maintaining the students’ satisfaction with the indoor environment they found in the United Kingdom is considerable because the indoor environmental quality is highly associated with the occupants’ health and wellbeing (Sakellaris et al., 2016). The degree of satisfaction, in particular with daylight conditions, greatly impacts individual mood, behaviour and cognitive performance (Wang and Boubekri, 2011). Therefore, gaining a better understanding of students’ daylight perception and expectations could increase their satisfaction with the indoor environment and also cognitive and academic performance. This knowledge can also be utilised by managers and daily operators of university buildings to help reduce the energy consumption of HVAC (Heating, Ventilation, and Air Conditioning) and illumination systems. For instance, a study on Korean office buildings showed that adjusting the indoor lighting conditions based on occupants’ expectations and utilisations helps to reduce lighting energy consumption by up to 43% (Yun et al., 2012). Moreover, it can support architects and lighting professionals working in the design of educational and residential buildings.

In order to maintain the satisfaction and academic performance of the students from different cultural backgrounds in the indoor environment they found in the United Kingdom, we needed to develop a methodology for assessing daylight perception. Therefore, this paper aims to (a) review the methods previously used to assess daylight perception and (b) establish a methodology for assessing daylight perception in the context of cultural background.

## The Human Response to Daylight: Evaluation Methods

In order to create a framework of the methodological approach to assess daylight perception in the literature, 482 research articles published in Scopus, Web of Science, and LEUKOS databases were searched for electronic records. The search was done in either title, abstract, or keywords of the papers using the following keywords: (Day)light perception, (Day)light expectation, (Day)light satisfaction, (Day)lighting sensitivity, (Day)lighting tolerance and (Day)light adaptation. The inclusion criteria were: (a) including at least one aspect of (day)lighting perception, (b) published in English, peer-reviewed journals excluding conference proceedings and books, and (c) published during any year from 1990 to November 2021. Relevant articles were classified depending on their methods and reported in Tables 1–3.

**Table 1:** The methods for circadian rhythm related assessment.

Method		References
**Cognitive performance**	**n-back test** *to measure working memory and working memory capacity*	Jaeggi and Jaeggi, 2011
	**CNV test** *to measure work performance with the average response times of correct answers* **Arithmetic task** *to reflect work performance with the ratio of correct answers*	Yasukouchi et al., 2019
	**Tsai Partington** *to evaluate the distributed visual attention* **d2 test** *to evaluate the sustained vigilance* **Baddeley test** *to evaluate the logical reasoning*	Chinazzo et al., 2019
	**Psychomotor Vigilance Test (PVT)** *including a Simple Reaction Time (SRT) test, a 2-Forced Choice Reaction Time (FCRT) test, and a Matching-to-Sample (MTS) test.*	Figueiro et al., 2011
	**Observation of Typing speed and accuracy**	Shamsul et al., 2013
	**Eye-tracking** *for measure numbers of fixation with a device such as Tobii® T60 Eye Tracker*	Shamsul et al., 2013
	**Class attendance** *as a measure of students’ performance*	Edwards and Torcellini, 2002
**Alertness**	**Visual Analogue Scale (VAS)** *to assess fatigue and alertness*	Karami et al., 2016
	**Karolinska Sleepiness Scale** *to measure both subjective sleepiness and alertness*	Shamsul et al., 2013; Chinazzo et al., 2019
**Sleeping pattern**	**Subjective sleepiness** *with some surveys such as the 9-item Karolinska Sleepiness Scale (KSS) and Sleep Habits Survey*	Figueiro et al., 2011; Jaeggi and Jaeggi, 2011; Figueiro et al., 2014; Karami et al., 2016; Choi et al., 2019
	**Sleep-activity behaviour** *A daily sleep-activity graph during the experiment*	Adamsson et al., 2018
	**Identification of morningness-eveningness** *Horne and Ostberg Morningness-Eveningness Questionnaire, Munich Chronotype Questionnaire (MCTQ) and Composite Scale*	Chung, 2009; Jaeggi and Jaeggi, 2011; Figueiro et al., 2014; Adamsson et al., 2018
**Mood**	Psychosocial stress *assessing with Perceived Stress Scale (PSS)*	Figueiro et al., 2011
	**Mood** *assessing with the Positive and Negative Affect Schedule (PANAS), and the Centre for Epidemiologic Studies Depression Scale (CES-D*)	Figueiro et al., 2011
	**Subjective general health evaluation** *using GHQ questionnaire*	Karami et al., 2016
	**Subjective mood and visual comfort** using *visual analogue scales (VASs)*	Choi et al., 2019

**Table 2:** The methods for subjective daylight assessment.

Method		References
**Interviews**	*Informal or semi-structured*	Axarli and Meresi, 2008; Dianat et al., 2013; Gentile et al., 2015; Yasukouchi et al., 2019
**Questionnaires**	**Questionnaire-based survey** *Snapshot subjective assessments such as Perceived lighting quality assessment and other created questionnaires mainly using a semantic differential method*	Cheung and Chung, 2008; Dianat et al., 2013; Gentile et al., 2015; Adamsson et al., 2018; Bournas et al., 2019; Chamilothori, 2019; Chinazzo et al., 2019
	**Questionnaire-based survey** *Long term subjective assessments*	Jakubiec et al., 2018
	**Subjective evaluations** *during experiments within different kinds of room (geometry/orientation/window type/façade type), different locations and different contexts (social or working context)*	Axarli and Meresi, 2008; Chamilothori et al., 2016, 2018, 2019; Bian and Luo, 2017; Jin et al., 2017
	**Visual comfort evaluation** *such as Visual comfort on visual analogue scales (VAS), Office Lighting Survey (OLS), Lighting Conditions Survey, NRC Canada Lighting Quality Scale, IEA retrofit monitoring user assessment survey, Indoor Environmental Quality Surveys*	Jaeggi and Jaeggi, 2011; Shamsul et al., 2013; Bian and Luo, 2017; Adamsson et al., 2018; Alicia et al., 2019
	**Indoor Environmental Quality Surveys** *such as Satisfaction with Environmental Features and Subjective ratings of discomfort glare (De Boer scale, Imperceptible-intolerable 4-point scale, Glare Sensation Vote, Visual comfort rating)*	Alicia et al., 2019
	**Other subjective measures of lighting** *Descriptive scales and Lighting preferences, beliefs, and behavioural consequences*	Alicia et al., 2019
	**Verbal questionnaire** *Evaluation of the impressions of how pleasant, interesting, and exciting the space*	Chamilothori et al., 2019
	**Questionnaires** *distributed by mail to evaluate brightness and distribution*	Bournas et al., 2019
**Quantification of daylight exposure consequently circadian light exposure**	**Actigraphy data from wearable biometric devices** *during the experiment with wristbands such as Empatica E4 wristband*	Figueiro et al., 2014; Chamilothori et al., 2018, 2019; Chinazzo et al., 2020
	**Actigraphy data from wearable biometric devices** *during the experiment with the Daysimeter*	Rea et al., 2010
	**Actigraphy data from wearable biometric devices** *during the experiment with the ambulatory circadian monitoring device (ACM)*	Arguelles-Prieto et al., 2019
	**Actigraphy data from wearable biometric devices** *prior to the study beginning, withbed and wake times with wristbands such as Actiwatch-L*	Chung, 2009;Jaeggi and Jaeggi, 2011; Yasukouchi et al., 2019
	**Actigraphy data from wearable biometric devices** *prior to the study beginning, with bed and wake times with the Daysimeter*	Figueiro et al., 2011
	**Asking time spent outdoors** *such as The Munich Chronotype Questionnaire (MCTQ), the Harvard Light Exposure Assessment questionnaire or self-prepared questions to get data about light exposure*	Adamsson et al., 2018
**Logs**	**Weekly log** *ratings of psychological well-being, daily sleep-activity and time spent outdoors*	Adamsson et al., 2018
	**Daily sleep log** *prior to the study beginning.*	Jaeggi and Jaeggi, 2011; Yasukouchi et al., 2019
**Seat preference**	**Surveys and observations** asking for the reasons for the choice of seat locations and direct observations of actual seating behaviour	Organ and Jantti, 1997; Kim and Wineman, 2005; Wang and Boubekri, 2010; Othman et al., 2012; Keskin et al., 2017; Gou et al., 2018
**Drawings**	**Drawing daylight boundary line** *between daylit and non-daylit area*	Reinhart and Weissman, 2012; Handina et al., 2017
**HDR-High dynamic image techniques**		Jin et al., 2017; Jung and Inanici, 2019; Chinazzo et al., 2020
**Daylight 3D renderings**	**Showing the renderings** *with the computer software of the same space to the subjects and ask to rate daylight composition*	Rockcastle and Andersen, 2015
**Immersive virtual reality (VR)**	**VR with headsets** *such as Oculus Rift CV1 and Oculus Rift DK2*	Chamilothori et al., 2016, 2018, 2019; Chamilothori, 2019

**Table 3:** The methods for objective measurements.

Method	References
**Heart rate (HR)** using some devices such as Empatica E4 wristband	Chamilothori et al., 2018; Chamilothori et al., 2019; Yasukouchi et al., 2019; Chinazzo et al., 2020
**Skin conductance (SC)** using some devices such as Empatica E4 wristband and Electrodermal activity (EDA) wristband	Chamilothori et al., 2018, 2019; Chinazzo et al., 2020
**Core body temperature** using some devices such as iButtons data loggers and wristband	Chung, 2009; Chinazzo et al., 2020
**Cortisol level** from salivary	Jaeggi and Jaeggi, 2011; Choi et al., 2019
**Melatonin secretion** from salivary, blood, urine	Figueiro et al., 2011; Jaeggi and Jaeggi, 2011; Tähkämö et al., 2015; Karami et al., 2016; Choi et al., 2019; Yasukouchi et al., 2019

### General Methodological Approach in the Reviewed Studies

Various methods have been developed and used to investigate how lighting conditions are consistent with human perception of daylight and daylight expectations. These methods have been applied in either real-world environments (Keskin, 2019) or laboratories under specified testing conditions (Chamilothori et al., 2016; Chinazzo et al., 2019; Yasukouchi et al., 2019).

Even though real-world environments provide an opportunity to conduct studies in a dynamic social context, people being observed cannot be tested under diverse environmental conditions. Conversely, participants in laboratory settings know they are the subject of study, which may affect their behaviour, making it challenging to associate results with real-life situations (Keskin, 2019). Nevertheless, laboratory studies enable researchers to investigate changes when daylight conditions are changed (Figueiro et al., 2011; Karami et al., 2016), which cannot be tested in real-world environment studies.

Although most methods and tools used in assessing daylight perception differ, their general methodological approach is similar; it combines subjective and objective measurements and assesses them depending on the existing lighting conditions collected by either spot measurements or daylighting simulations. The studies are also often supported by circadian rhythm parameters, such as cognitive performance, alertness, sleep quality, and mood. Nevertheless, almost all studies have used one or more methods to assess the changes occurring in daylight perception concerning the variation in the luminous environment.

### Methods Regarding Circadian Regulation

Circadian rhythms are approximately 24-h cycles controlled by an internal master clock in the brain responsible for regulating many physiological (body temperature and hormones) and behavioural (sleep, mood, alertness and performance) changes (Skene and Arendt, 2006). Circadian rhythms are mainly affected by the intensity and timing of light exposure (Arguelles-Prieto et al., 2019) and adjusted at regular intervals by receptors transmitting non-image-forming information of light, which activate the circadian system (Bellia et al., 2011).

Exposure to a high amount of daylight (for example, spending a large amount of time outside or sitting indoors by a big window) has been shown to be related to enhancer effects in students’ cognitive and academic performance (Shishegar and Boubekri, 2016). Previous research that examined the impact of different shading systems on cognitive function performance, satisfaction, and eyestrain in a living lab has also established that satisfaction with indoor daylight conditions could result in higher cognitive performance (Jamrozik et al., 2019). Most researchers have benefitted from commonly used tests and techniques such as the Psychomotor Vigilance Test (PVT), usually used to assess the link between daylight and cognitive performance. Others have also used class attendance or typing speed and accuracy as an indicator of cognitive performance.

On the other hand, several studies have proved that daylight exposure significantly influences occupants’ mood state (Boyce et al., 2003). Küller et al. (2006) indicated that the participants’ mood reached the lowest level when describing the daylight conditions as too insufficient. Specified scales (PSS, PANAS, CES-D and VASs; Figueiro et al., 2011; Choi et al., 2019) and questionnaires (GHQ; Karami et al., 2016) are usually utilised to investigate the association between the exposed daylight conditions and mood states.

Changes in circadian rhythms have also been associated with sleep quality and alertness in addition to mood and cognitive performance (Garbarino et al., 2020). The Karolinska Sleepiness Scale (KSS) has been mainly used to measure both subjective sleepiness and alertness (Shamsul et al., 2013; Chinazzo et al., 2019). Tools such as the Horne and Ostberg Morningness-Eveningness Questionnaire and the Munich Chronotype Questionnaire have also been used to assess the sleep quality of participants grouped according to their sleep–wake behaviour (morningness–eveningness; Jaeggi and Jaeggi, 2011; Adamsson et al., 2018).

### Physiological Biomarkers as a Consequence of Exposure to Daylight

Physiological measurements (biomarkers) are regarded as indicators of previous light exposure; in other words, how much a participant was exposed to light during a specific time. The duration, timing and intensity of exposed daylight may affect people’s satisfaction with current daylight conditions and the regulation of their circadian rhythms. Thus, the assessment of physiological biomarkers could play a crucial role in assessing and interpreting an individual’s daylight perception.

The objective measurement of daylight perception considers the assessment of physiological biomarkers such as heart rate (Chamilothori et al., 2019; Chinazzo et al., 2020), skin conductance (Chamilothori, 2019; Chamilothori et al., 2019), core body temperature (Chung, 2009; Chinazzo et al., 2020), cortisol level (Jaeggi and Jaeggi, 2011; Choi et al., 2019), and melatonin secretion (Figueiro et al., 2011; Jaeggi and Jaeggi, 2011). Heart rate, skin conductance, and body temperature have been measured using wristbands, while melatonin secretion is measured using either salivary, blood, or urine samples.

### Subjective Assessment of Daylight

Since individuals are physically and psychologically influenced by daylight (Chung, 2009), objective measurements should be complemented with subjective evaluations. However, some studies (Galasiu and Veitch, 2006; Bellia et al., 2017; Lo Verso et al., 2021) have shown that correspondence between exposed daylight conditions and subjective assessment of the occupants is not always observed because of individual differences. Subjective assessment methods mainly use questionnaires to obtain information through semantic differential techniques, measuring the participant’s overall reaction to specific factors such as ambient illumination of different light sources or horizontal illuminance and brightness of a space (Jin et al., 2017; Albertazzi et al., 2018). Similarly, open-ended questions are used to gain deeper and new insights into the feelings towards daylight conditions, for instance, asking how participants describe the lighting conditions and how they feel under those conditions. Information is usually collected concerning the participants’ background (age, gender, work schedule, sleep and wake times, previous daylight exposure etc.), their evaluation of daylight illuminance and distribution, and their general satisfaction with the indoor environment (Levin, 2017).

As a method for assessing previous daylight exposure, questionnaires require participants to estimate the frequency of exposure to daylight in a particular period (Adamsson et al., 2018). For instance, the Munich ChronoType Questionnaire (MCTQ) involves estimating the time spent outdoors on workdays and free days, assuming regular light exposure patterns. Likewise, the Harvard Light Exposure Assessment questionnaire (H-LEA) emphasises the importance of time duration and period of light exposure during the daytime to various artificial and natural light sources. Information about previous daylight exposure is also collected with the use of devices that participants are asked to wear, for example wristbands, Daysimeter and ACM (Chamilothori et al., 2019) before (Figueiro et al., 2011) and/or during the experiment (Rea et al., 2010). The collected data is often supported by self-written logs (Adamsson et al., 2018). These devices are also used to gain insight into the activity and sleep pattern of the participants and the amount of daylight they were exposed to.

Few researchers have preferred other subjective methods such as interviews to test the influence of different daylighting configurations on participants’ daylight perception (Dianat et al., 2013; Gentile et al., 2015). Moreover, the use of evaluation techniques, such as seat selection, have been applied, where it has been assumed that daylight perception and expectation are associated with seat preference and window location (Wang and Boubekri, 2010; Keskin et al., 2017). In this case, the selected desk’s illuminance level could be used as an indicator of daylight perception. Additionally, a unique method was proposed by Reinhart and Weissman (2012) and also used by Handina et al. (2017), given its potential as a representation tool of how daylight composition can be perceived in a space. Handina et al. (2017) have considered the daylight boundary line method to assess perception through the definition of daylit and non-daylit areas drawn by participants. In this methodology, participants have been required to draw a line whenever they notice a boundary between brightness and darkness in the experiment room. Their initial results showed that the percentage of the area enclosed with the contour line of DA300 lx, 50% (illuminance level of at least 300 lux over at least 50% of the space) in the observed space (55%) is close to the partially daylit area (56%), which is the area perceived as bright by at least 25% of participants. Furthermore, high Dynamic image techniques (Jung and Inanici, 2019) and 3D daylight renderings (Rockcastle and Andersen, 2015) have also been used to evaluate the human perception of the daylight composition found in shown scenes. In the further development of these techniques, subjective daylight perception under various computer-generated conditions has been assessed using scenes displayed with the Immersive virtual reality (VR) technique (Chamilothori et al., 2016).

## Methodology

Fifty MSc students were brought all together to the Bartlett Library, asked to complete a questionnaire before the experiment and undertake a set of tasks while going around the library. The library was assessed during one of the sunniest days in December 2019 (between 13:00 and 14:00); a day with a clear sky was selected to get maximum daylight throughout the library during the experiment. The day and time of the study were decided based on both the previous years’ daylighting data obtained from Public Health England and weather data from the Met Office. All tasks took between 20 and 25 min to complete. Collected subjective responses from participants were evaluated depending on the daylight availability of the room obtained from a lighting simulation tool.

As previously highlighted, the effect of lighting conditions on human perception and expectations should be investigated using objective measurements and subjective evaluations. However, only subjective evaluation methods with different applications could be utilised to complement each other for situations where a considerable amount of data collection from objective measurements may not be feasible and accessible. Thus, in this study, only these subjective evaluation methods were applied; seat preference, subjective ratings and daylight boundary line drawings.

### Participants

An invitation to participate in the study was sent *via* email to 348 postgraduate students enrolled in MSc programs at the Bartlett School of Architecture, UCL. Seventy-six responded that they would be happy to be involved in the experiment, but only 50 students (15 males/35 females) aged 20–34 years old were recruited for this study.

In terms of cultural background, the ethnicity of participants and the time spent in London were considered. Eleven participants (22%) described themselves as White, whereas 33 students (66%) stated they have an Asian background. Only five participants (10%) defined their ethnicity as other ethnic backgrounds. Most of the students (72%) were overseas students who had spent less than 3 months in London.

### Field Site

**Figure 1:**
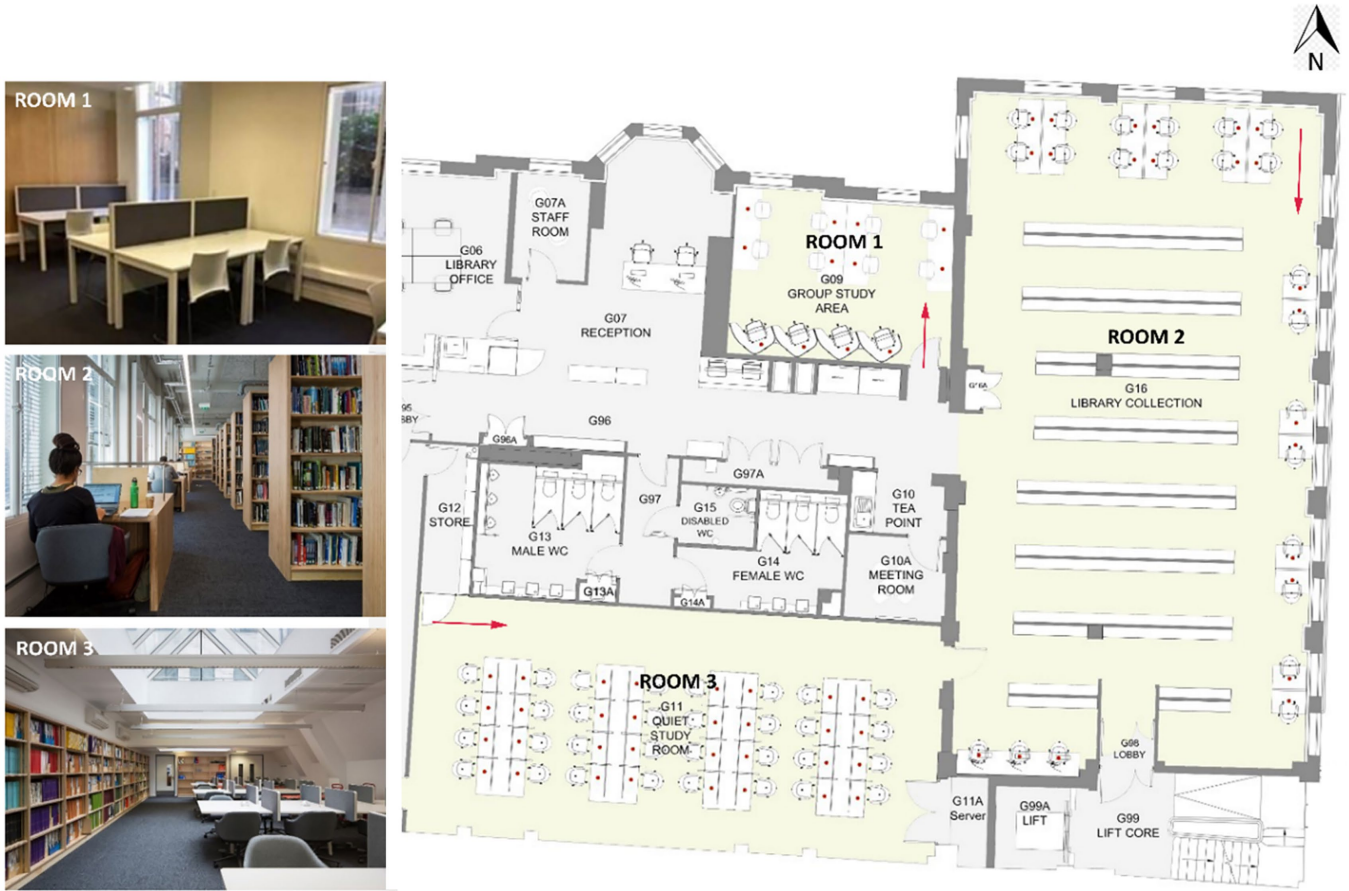
Plan of the Bartlett Library (The red arrows represent the locations from where the photos on the right side of the figure were taken).

The study was carried out in the UCL Bartlett library located on the ground floor of a six-storey building. The library comprises three main study areas (Figure 1). The group study area (Room 1) accommodates eight shared desks and four individual cubicles and has two side windows in the north-facing external wall; the library collection area (Room 2) has 12 shared desks and 11 individual desks and several side windows facing north and east orientations; the quiet study room (Room 3) is an open-plan space with a skylight, and 32 shared desks. Details of the rooms and technical properties of the surfaces are illustrated in Appendix 1.

### Quantification of Daylight Availability in the Library

Parametric modelling and daylight simulations were used to get information concerning daylight availability at desks in each room at the library. Spot illuminance measurements were also used to calibrate the created model. AutoCAD and Rhino were used to produce 2D and 3D drawings of the library. Then, Grasshopper was used to create parametric modelling for lighting performance analysis with Ladybug and Honeybee plugins.

Previous studies show that computer predictions with simulation methods demonstrate higher accuracy than measurements taken in real-world conditions. The simulation method results involve an acceptable amount of error arising from either unpredictable sky conditions at that moment or the incorrect input parameters in the simulation model. Therefore, it is always more reliable to compare daylight performance predictions obtained from computer simulations with physical measurements taken in the real space. Since it demonstrates how much simulation results correspond to actual daylight conditions. Daylight modelling built-in Radiance was validated against actual illuminance measurements at a specific point, date and time. A strong association between simulation results and actual daylight measurements was found (*p* < 0.05, *R*^2^ = 0.89). In other words, the difference in values between spot measurements and simulation results are negligible, and simulation results represent the real daylight illuminances with an acceptable error range.

### Contribution of Electric Light to Total Illuminance

On the day in which the study was performed, students were exposed to electric light in addition to daylight.

The contribution of electric light to total illuminance was investigated by measuring the electric light illuminances in the middle of each desk using a Konica Minolta Illuminance meter T-10A on the 30 November 2019 between 16:45 and 17:15 after sunset. Thereafter, these illuminances were compared with total illuminance measurements taken during the experiment. The electric light illuminance values on the work planes were found highly correlated with the total illuminance measurements (*p* = 0.001). For this reason, it was assumed that all desks receive the same amount of electric lighting, and therefore variations between them would be due to daylight alone.

### Subjective Daylight Assessment Methods

#### Questionnaire Design

A questionnaire was designed to include the three methods used in this study: seat preference, subjective rating, and daylight boundary line drawings. The questionnaire contained multiple-choice, Likert scale, and open-ended questions and was divided into five sections; the first two sections of the questionnaire were completed by participants before entering the library and considered information regarding (1) demographic; gender, and age, (2) time spent in London (months). The following three sections considered specific questions and tasks related to the methods explored to measure participants’ daylight perception; (3) seating preference and reasons for seat selection, (4) evaluation of daylight availability at the best seat selected, and (5) differentiation between daylit and non-daylit spaces (boundary line drawing). The procedure order was specifically designed to start with open questions regarding seat preference, and after then daylight specific questions to lead on to influence the participants’ responses, thus the latter questions would not impact the responses to the former ones. Ethical approval for this study was obtained from the UCL Research Ethics Committee in November 2019.

#### Task 1: Seat Preference

Seating that meets students’ needs and preferences could promote a longer stay in the libraries and keep students motivated, influencing their emotions and learning abilities. Many disciplines have extensively discussed the influential factors on seat preference in a learning environment. It has been shown that the affecting factors arising from the physical environment that govern the decision of seat selection are daylight (Othman and Mazli, 2012; Keskin et al., 2017), ambient temperature, type of furniture, proximity to other occupants (Dubois et al., 2009), quietness, outdoor view, privacy, social interactions such as close to friends, entrance or circulation (Gou et al., 2018), students’ degree of territoriality and seat arrangements (Kaya and Burgess, 2007).

Even though the importance of daylight on seat preference varies from study to study depending on the function of the room, time interval, time of the day and year (Keskin, 2019), some researchers have proved that daylight is the most important reason for seat selection (Alicia et al., 2019; Izmir Tunahan et al., 2021a,b) and the most frequently chosen as a reason for seat selection (Keskin, 2019). Hence, in this study, it was assumed that seat preference could be used to understand whether participants valued the daylight component. The daylight availability of the selected desk was then considered to be an indicator of the daylight conditions the participant prefers. For this purpose, participants were asked to indicate the three best and the three worst seat locations from the library’s seating plan, and within those categories, the most and least liked. They were also asked to specify the reasons for their selection to examine whether the selected desks (best and worst) coincide with those where daylight levels were high and low, respectively, hence if the daylight component is an influential factor when deciding where to sit.

#### Task 2: Subjective Ratings

The subjective rating method involves asking participants to describe the daylight conditions on a specific desk surface. This method has been utilised in many lighting studies, and many researchers have found participants’ own perceptual statements compatible with actual daylight conditions. However, subjective evaluations may not represent daylight availability completely because of individual differences in some cases.

**Figure 2:**
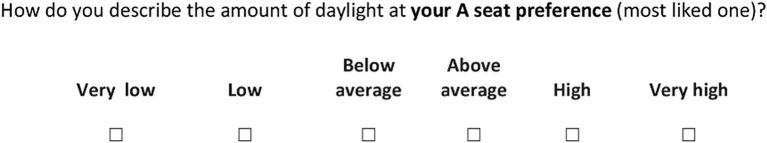
The question regarding subjective ratings.

This method was applied to determine the degree to which subjective statements represent daylight availability in a space and investigate whether people perceive daylight conditions in line with actual measurements. The possible reasons causing the variation between actual measurements and people’s perceptions could help identify ways to increase occupant satisfaction in the built environment. For this purpose, participants were asked to describe the amount of daylight at the best seat they have selected using a six-option scale derived from the BUS questionnaire (Leena, 2017; from very low to very high; Figure 2). Thus, daylight availability at a specific desk was tested depending on how participants perceived it.

#### Task 3: Daylight Boundary Line

**Figure 3:**
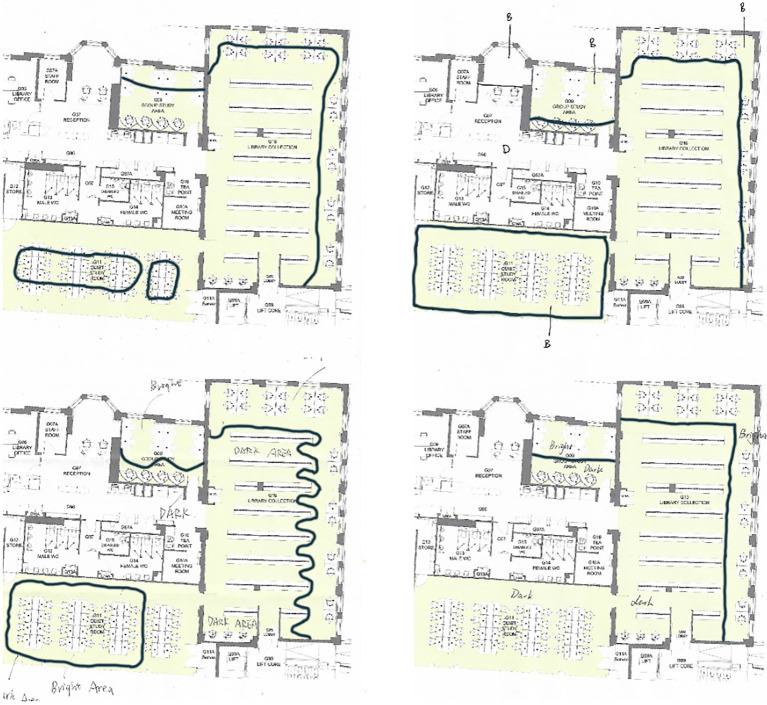
A few examples of participants’ drawings in response to the question asking them to draw a boundary line between daylit and non-daylit spaces.

This unique method proposed by Handina et al. (2017) was used given its potential to represent how daylight composition can be perceived in a space. For this purpose, participants were instructed to draw on a copy of the library floor plan, ‘daylight boundary lines’, whenever a significant change of contrast was found or a bright area was perceived when moving around the library (Figure 3). The drawn boundary lines were then scanned and overdrawn in AutoCAD to overlay the perceived bright areas, which were assumed to indicate the perception of adequate daylight in this study. Finally, all drawings were superimposed on top of each other and evaluated based on daylight availability at a specific time.

#### Methods of Analysis

All the statistical analyses were conducted using the software package SPSS 20.0. Univariate descriptive statistics (response frequencies, means, and standard deviations) were calculated for each variable. Evaluations of the data obtained from three subjective methods were carried out separately as described below.

##### Analysis of Seat Preference

Initially, influential reasons for the best and worst seat selections and the importance of daylight in the selections were considered. Secondly, daylight availability at the best seat selected was evaluated using ordinal regression. Lastly, the best and worst seat selections were evaluated on the seating map concerning other influential factors on seat selections apart from the contribution of daylight.

##### Analysis of Subjective Ratings

Subjective ratings were evaluated based on the perceived daylight conditions towards daylight availability at the best seat selection using an independent-samples *t*-test.

##### Analysis of Daylight Boundary Line Drawings

Daylight boundary line drawings were assessed with the methodology created by Handina et al. (2017). Initially, the variation in participants’ perceived bright area was analysed using descriptive statistic methods. Secondly, the statistical quartile concept was used to categorise and visualise the areas agreed by a certain number of participants as bright. Spaces were differentiated as *fully daylit* (area agreed as bright by at least 75% of the participants), *partially daylit* (area perceived as bright by at least 25%) and *non-daylit* (area perceived as bright by less than 25% of participants). Lastly, categorised areas representing the participants’ overall daylight perception were overlapped with daylight availability to investigate if they correspond with each other.

##### Analysis of Daylight Simulations

Data obtained from seat preference and subjective rating methods were evaluated based on point-in-time climate-based calculations positioned horizontally in the middle of each working desk, which has been found to have a better association with seating behaviour than other daylight metrics for predicting daylight availability in previous studies (Keskin et al., 2015). Daylight boundary line drawings were assessed using DA300lx,50% (50% of the occupied time when the target illuminance of 300 lux on a horizontal plane is met by daylight) because of a more robust association with the daylight composition of space than others (Handina et al., 2017).

## Results and Discussion

### Seat Preference

#### Reason for Seat Selection

Participants were instructed to select the three best and the three worst seats and indicate the reasons for their selection in an open-ended question. Each participant stated at least one reason for their seat selection (Table 4). The number of reasons stated for the seat selection was greater than the number of respondents who answered the question. This caused the total response percentages to exceed 100%.

**Table 4:** Participants’ responses concerning the reasons for choosing the best (left) and worst (right) seats in the library.

Reason for best seat selections	A best (%)	B second-best (%)	C third-best (%)	Reason for worst seat selections (%)	1 worst (%)	2 second-worst (%)	3 third-worst (%)
**Quietness**	14.3	4.0	7.7	**Noisy**	19.2	12.5	13.0
**Natural light**				**Natural light**			
Daylight	53.6	44.0	57.7	Lack of/insufficient daylight	61.5	62.5	52.2
Skylight	10.7	24.0	3.8	Skylight	3.8	4.2	4.3
Proximity to window	14.3	12.0	15.4	No window	0.0	4.2	4.3
**Outdoor view**	25.0	4.0	15.4	**Lack of/ unpleasant outdoor view**	11.5	12.5	13.0
**Privacy**				**Privacy**			
Privacy	32.1	20.0	11.5	No privacy	7.7	8.3	0.0
Private position	7.1	8.0	0.0	Non-private position	0.0	0.0	4.3
Feeling isolated	0.0	24.0	11.5	Feeling isolated	7.7	8.3	8.7
**Desk**				**Desk**			
Desk feature	7.1	4.0	15.4	Desk feature	0.0	4.2	13.0
Desk location	3.6	8.0	15.4	Desk location	23.1	12.5	8.7
**Indoor conditions**	7.1	4.0	3.8	**Indoor conditions**	15.4	12.5	21.7

Daylight was the most dominant reason when selecting the most liked desk, followed by privacy, outdoor view and quietness, respectively. These results align with the findings of Dubois et al. (2009); daylight was the most significant reason for seat selection. Keskin (2019) also reported daylight as a highly mentioned reason for seat selection in their experiment. In other respects, indoor conditions such as temperature and air quality were other influential parameters for seat selection. Other reasons mentioned related to specific desk features were wideness, proximity to the circulation route or entrance, enabling to study individually or with friends, being at the corner or the back of the room and access to facilities such as a computer or plug socket. The worst seats were also associated with unsatisfactory daylight conditions; and with distractive noise, lack of or unpleasant outside view and non-private environment.

Although, in general, participants seem to agree on the reasons given when selecting the best and worst seats, there were a few cases where a particular desk was selected as the worst and the best by participants. Although seat preference varied from person to person depending on individual needs and expectancies, the majority of the participants considered it important to have a satisfactory daylighting level, face the least people, and have an outdoor view of greenery.

#### Daylight Availability at the Best Seats Selected

The daylight availability at the best desks selected by participants showed that 44% of the participants (*N* = 22) described the amount of daylight on their best desk as very high, 42% (*N* = 21) stated that the daylight conditions were high, and 6% (*N* = 3) as above average. In contrast, only 8% characterised the daylight conditions as low or very low. These results support the idea that most people prefer desks with a high amount of daylight, which could be with/without consciousness (Kahneman, 2011). Since the awareness of our behavioural responses to the physical environment is limited, and some of our behaviour is not under our conscious control.

An independent-samples *t*-test was also carried out to check whether there was a significant difference in daylight illuminance level of the best seats selected between participants who indicated daylight as the reason for their selection and those who did not. The findings showed that people who mentioned daylight as a reason preferred the desks with much higher daylight illuminance levels (468.5 ± 437.1 lx) than those that did not mention daylight as a reason (174.9 ± 183 lx), [t(48) = 2.1, *p* = 0.052]. It could be explained that daylight availability on a specific desk that meets an occupant’s needs and preferences, namely individual daylight expectation, usually influences seat preference. Therefore, daylight availability of the selected desk could be used as an indicator of an individual’s daylight preference.

#### Other Influential Factors

**Figure 4:**
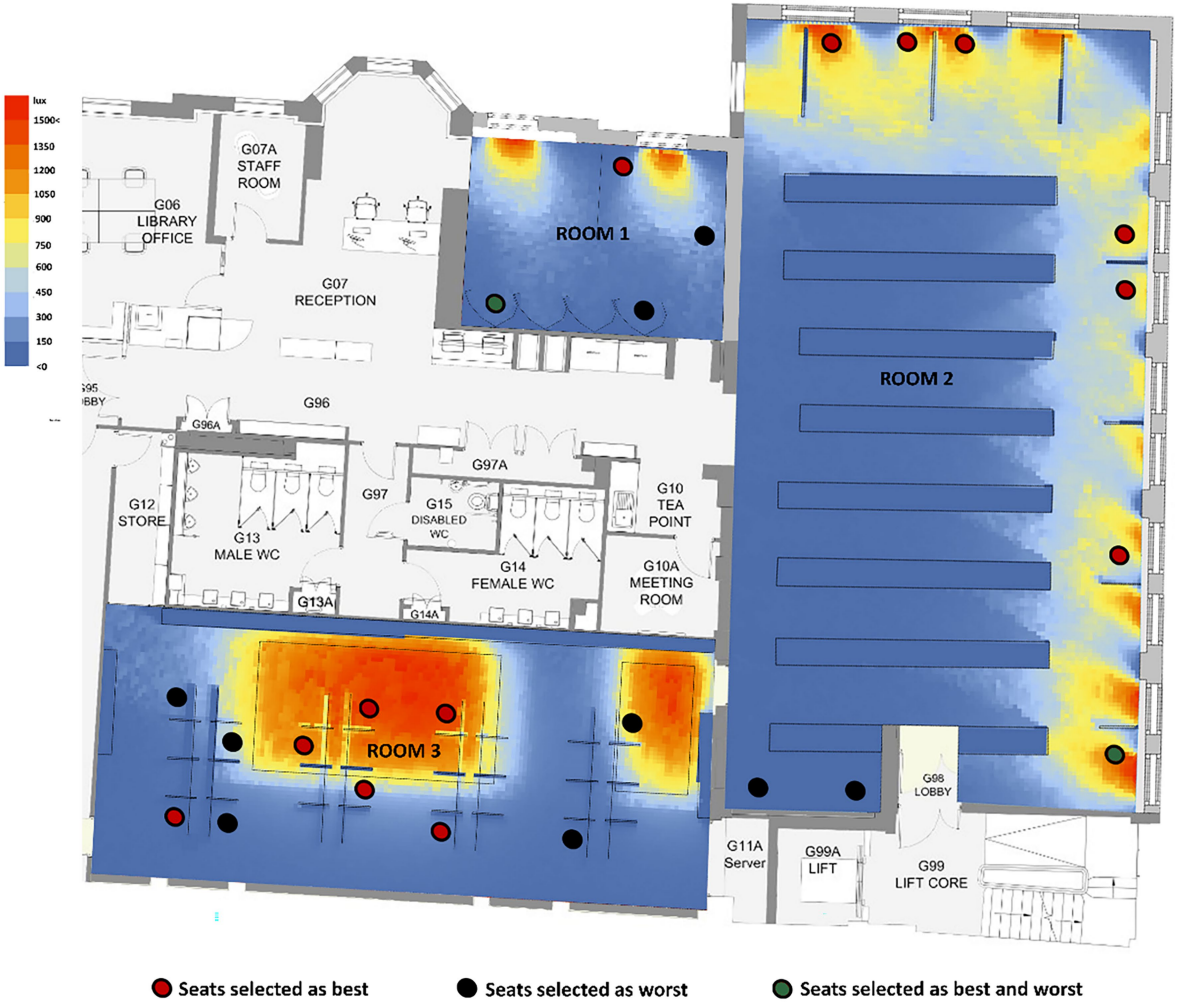
Best and worst seat selected by participants against daylight availability.

Figure 4 presents the seat preference configuration against the library’s daylight availability when the experiment was conducted. The categorisation of lighting levels was done based on the recommended range for library reading rooms (between 300 and 500 lux; Keskin, 2019). It can be seen that most (86%) of the seats selected as the best are located in areas with high illumination, whereas most unpopular desks are located in places with poor or lack of daylight. Interestingly, two desks were regarded as both best and worst by different participants. One of them, located in Room 1, corresponds to an individual cubicle that does not have access to outdoor view or acceptable daylight levels. The desk was selected as the worst seat by a participant because of the deficient daylight level; however, another participant preferred it because the desk was at the corner and more private than others. Another desk, described as both best and worst by five participants, is located near the window and in the corner of Room 2. The desk has a satisfactory level of daylight and outdoor view of greenery, which some participants positively appraised; however, others were negatively affected, given its closeness to an emergency exit and facing the people passing through the circulation route.

Desks in Room 3 under the skylight had a high level of daylight when the study was conducted; however, they were not preferred as expected. The desks near the window in Room 2 were more popular than the desks in other rooms. Six participants stated that they do not feel comfortable in the open-plan layout of Room 3, even though it has excellent daylight levels, especially at some desks. They also mentioned that their screens were visible to other students and that even if it was a silent room, it was easy to get distracted due to facing other people. These findings emphasised that seat preference cannot be examined only in relation to daylight, and it should be investigated together with other components reported in the study such as privacy, outdoor view and quietness.

The role of daylight on seat selection may also vary depending on the context, sample characteristics, and the activities participants are requested to undertake. For instance, this study’s results could have been different if the participants were in real need of using the space for their respective studies (e.g., reading and writing for an assignment). In that case, privacy and quietness could have been more important than natural environment components such as temperature, lighting and outdoor view. Therefore, the study design might have affected the participants’ natural environmental attention and evaluation of the space and desks. However, although the importance of daylight varies from study to study, it always remains an essential factor for seat selection.

### Subjective Ratings

After selecting the best and worst seats, participants were asked to rate the daylight conditions on the work plane at the seat they had selected as the best in the library. Then, the perceived daylight conditions of the participants were evaluated towards daylight availability at the best seat selection using an independent-samples *t*-test. Although some individuals described the amount of daylight different from actual measurements, it was assumed that the contribution of daylight to horizontal illuminance on the desk had a significant effect on the subjective assessment of daylight, *p* = 0.002. The correspondence between subjective ratings and daylight measurements proved that subjective rating is a suitable method for evaluating daylight perception and vice versa. However, even if the difference between the subjective ratings and daylight conditions was minimal, inter-individual differences in perceiving daylight conditions need further investigation.

### Daylight Boundary Line

#### Variation in Perceived Daylight

**Figure 5:**
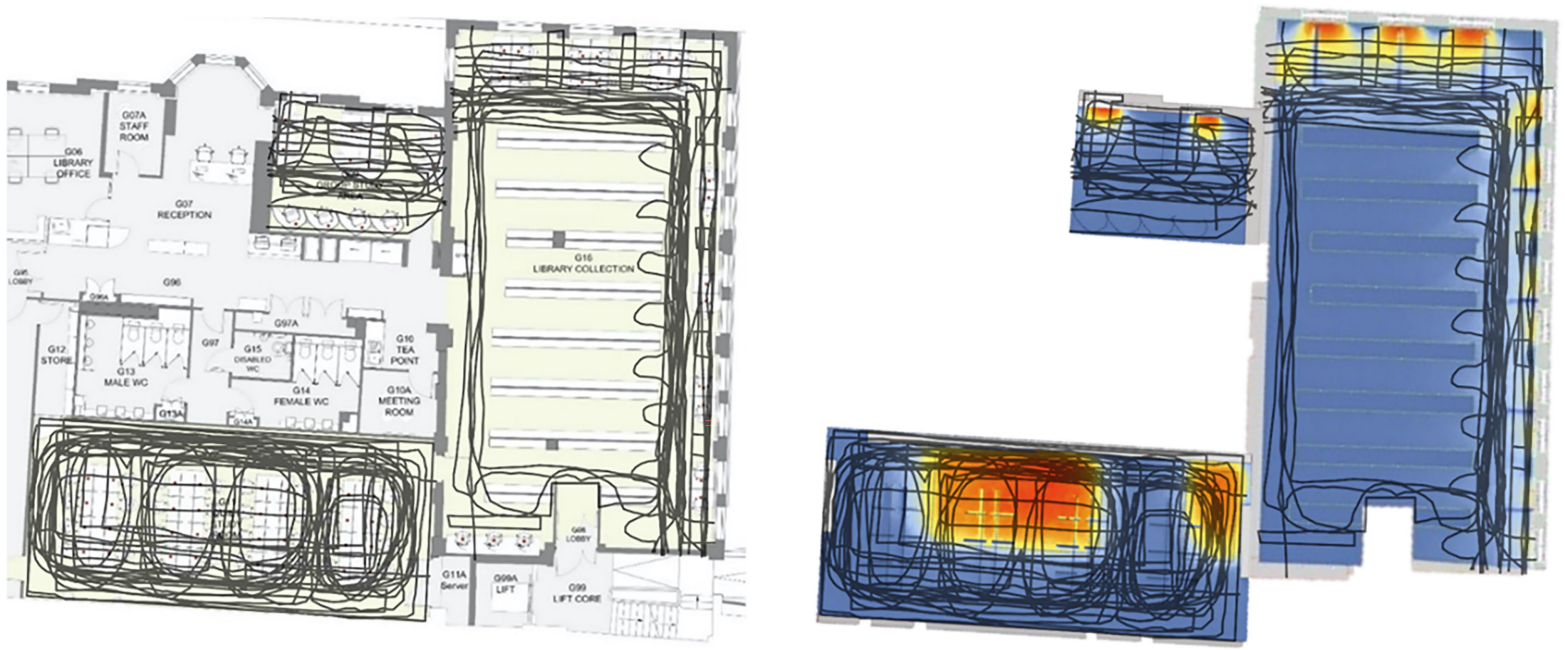
Daylight boundary line drawings of the participants (left), Comparison of drawings with daylight availability (right).

The library’s indoor daylight conditions were assessed by asking participants to draw a boundary line when they noticed a distinction between daylit and non-daylit spaces. A few examples of participants’ drawings are shown in Figure 3. In this experiment, some participants described the daylight availability in certain areas as very high, whereas others found the daylight in the same areas low or insufficient. The overlapped drawings gathered from all participants are presented in Figure 5. Participants’ average perceived bright area in the library varied from ~16 to ~100 square meters (mean = 40.3, SD = 24.6, *N* = 50). Perceived daylight conditions varied over an extensive range from person to person regardless of actual daylight measurements. Therefore, aspects that can intervene and cause the discrepancy between actual daylight measurements and participants’ perceptions from drawings deserve further attention.

#### Daylight Availability and the Overall Perception

**Figure 6:**
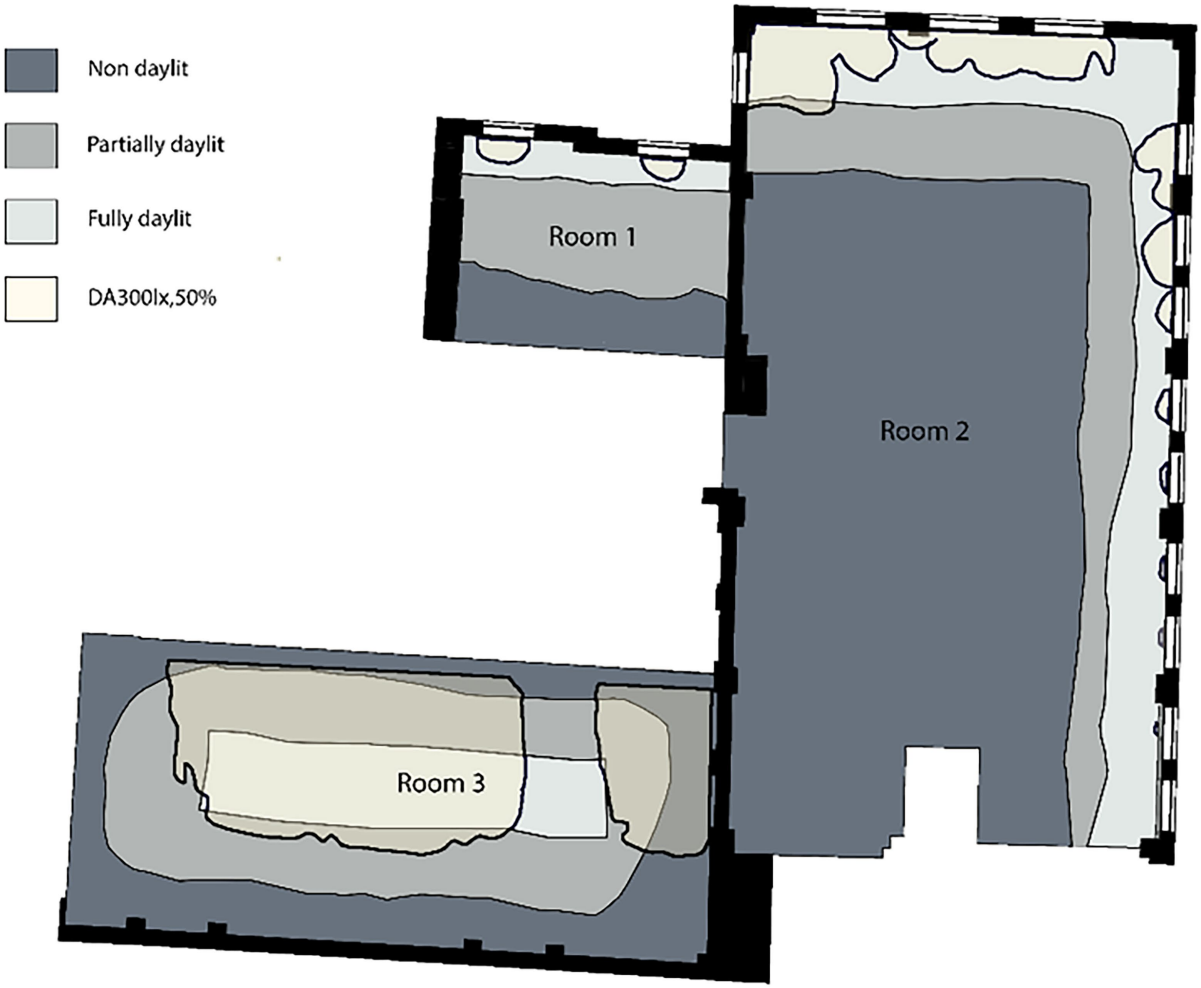
Comparison of the overall daylight perception with percentage of the area enclosed with the contour line of DA300lx,50%.

In order to categorise and visualise the areas agreed by a certain number of participants as bright, the overall perception of daylight composition within each room was evaluated using the statistical quartile concept. Spaces were differentiated as *fully daylit* (perceived as bright by at least 75% of participants), *partially daylit* (perceived as bright by at least 25% of participants), and non-daylit (area perceived as bright by less than 25% of participants; Figure 6). Despite the inter-individual differences in the participants’ perceived daylight conditions from drawings, there are still apparent areas in the centre of rooms 2 and 3 that all participants agreed to be the dimmest and brightest, respectively.

The participants’ overall daylight perception was overlapped with daylight availability in the library to determine the difference between perceived daylight availability from drawings and actual daylight measurements. Handina et al. (Handina et al., 2017) found that the most compatible metric to evaluate boundary line drawings concerning daylight availability in a space is DA300lx,50%. Thus, Daylight Autonomy (300 lx,50%) was used to evaluate daylight boundary line drawings as a daylight availability metric that corresponds to 50% of the occupied time when the target illuminance of 300 lux on a horizontal plane is met by daylight.

As seen in Figure 6, only in Room 3, the percentage of the area enclosed with the contour line of DA300lx,50% (41.3%) to some extent, corresponds to the partially daylit area (45.1%). However, the percentage of DA300lx,50%, was not close to the other two rooms’ fully daylit areas. Therefore, this method could somewhat explain the tendency in daylight perception of a group of people, despite the noticeable inter-individual differences in the daylight boundary line drawings. It could be useful for comparing daylight perception of a particular group of people, such as people’s perceptions living in different latitudes. However, space’s characteristics such as the room’s size, window type and size, and seat configuration could explain the possible difference in participants perception. Also, as seen in Figure 5, the degree of agreement in the participants’ perceived bright area varied. Even though perceived bright areas varied from person to person in Room 2 and 3, the agreed daylit space was more noticeable. Perceived bright areas in Room 1 varied on a wide range, and there is no agreement in the participants’ perception. These findings agree with Handina et al.’s (Handina et al., 2017) work, where a noticeable difference was found in the subjective daylight evaluations between small and large spaces. Overall, these findings indicate that this method could be used to compare the overall daylight perception of a particular group of people; however, it needs further investigation for the individual assessment of subjective daylight.

### Initial Findings From the Developed Methodology

This paper aims to review the methods previously used to assess daylight perception and establish a methodology for assessing daylight perception in the context of cultural background. As mentioned in the results section, seating preference and subjective ratings seem as suitable methods for evaluating daylight perception of individuals. Therefore, as a part of cultural background in the lit environment (Izmir Tunahan et al., 2021), the contribution of ethnic background and time spent in a specific environment to the participants’ responses was analysed. The results from the seat preference method showed that when selecting the best seats, the leading reason for 48.5% of Asian participants was daylight, followed by privacy (15.2%), quietness (6.1%) and indoor conditions (6.1%). On the other hand, 33.4% of White participants selected their favourite desks considering daylight as a priority. Subjective rating method results also showed that Asian participants described daylight conditions on the best-selected desks as equal or lower than actual measurements. In contrast, White participants described daylight conditions as similar or higher than actual daylight conditions. This finding shows similarity with Lee and Kim’s (Lee and Kim, 2007) study, which showed that Asian people felt more comfortable than Caucasians towards high glare levels of luminance.

In terms of time spent in London, study findings showed that participants that had been in London for longer periods gave less weight to daylight while selecting a seat than students that arrived a couple of months before the study. Four students born and grew up in London preferred desks with significantly less daylight than non-Londoners. In parallel with their seating preferences, students who spent more time in London described the daylight conditions at the best desk as more acceptable. Acclimatisation to daylight conditions over time could affect subjective daylight evaluations and explain this finding just as shown by Martin et al. (Hébert et al., 2002). However, participants’ daily routine, how long they are exposed to outdoor daylight conditions and in which timeframe also matter in addition to the daylight availability of the city. Together these findings show that there could be an association between cultural background and subjective daylight evaluations; however, it needs further investigation with a large sample size of participants considering all cultural background components.

### Limitations and Future Work

- The study was limited to a particular place and a particular group of people at a given point in time. The small sample size was another limitation that did not allow to generalise of the findings.
- The role of daylight on seat selection may vary depending on the context, sample characteristics, and the activities participants are requested to undertake. Study results could have been different if the participants were in real need of using the space for their respective studies (e.g., reading and writing for an assignment). In that case, privacy and quietness could have been more important than natural environment components such as temperature, lighting and outdoor view. Therefore, the study design might have affected the participants’ natural environmental attention and evaluation of the space and desks.
- Even if the difference between the subjective ratings and daylight conditions was minimal, the reasons for perceiving daylight conditions different from other individuals need further investigation, and inter-individual differences should be examined deeply in further studies.
- The use of drawings to measure participants’ perceptions, such as the daylight boundary line method, has some limitations because it involves cognitive and motor processing simultaneously. Therefore, it is suggested (Mitchell et al., 2011) that when a drawing is used as a research method, it should entail participants’ drawing and talking, or drawing and writing to interpret the meaning embedded in their drawings.
- The impact of cultural background on daylight perception was evaluated considering only ethnic background and time spent in London. However, cultural background in the lit environment comprises many aspects. Further analysis is needed as suggested by (UKCISA, 2017) considering the luminance environment where people used to live, the luminance environment they were recently exposed to, the socio-cultural background, and individual lifestyle daily routines.

## Conclusion

Daylighting is an essential component of the indoor environment that can greatly influence the occupants’ comfort and wellbeing. For assessing the daylighting quality, photometric measurements on their own do not wholly represent the subjective aspect of the lighting environment; therefore, more attention should be paid to how participants perceive the same daylight conditions and which method can predict the daylight perception of the participants much better. This paper has presented a summary of current methods for assessing daylight perception and established a methodology for assessing daylight perception in the context of cultural background. In lighting studies, culture represents the many aspects from individuals’ characteristics and the climatic and indoor conditions people have experienced. Hence, people from different cultural backgrounds might have different expectations of the lit environment. This knowledge could be used to investigate how users interact with the building and develop strategies to reduce unnecessary electricity consumption in addition to the contribution to human health and wellbeing.

This paper showed that subjective ratings, the amount of daylight described by participants, coincide with the daylight availability on specific surfaces. However, there remains a slight difference between participants’ statements and actual daylight conditions. The reasons why daylight conditions are perceived differently by participants need further investigation. The findings from the seat preference method showed that daylight was the most dominant reason when selecting the best desks in the library, followed by privacy, outdoor view and quietness, respectively. Although the reasons for seat selection varied, the majority of the participants agreed on particular reasons; satisfactory daylighting level, facing the least people, and a greenery outdoor view. This study also showed that the perceived daylight conditions obtained from the daylight boundary line method varied extensively from person to person regardless of actual daylight measurements. Therefore, aspects that can intervene and cause the discrepancy between actual daylight measurements and participants’ drawings deserve further attention. Initial results from the developed method demonstrated that there could be an association between cultural background and subjective daylight evaluations; however, it needs further investigation with a large sample size of participants considering all cultural background components.

Together these findings showed that subjective rating and seat preference methods could be used to evaluate daylight perception. Although daylight availability corresponds better with subjective statements, collecting participants’ subjective responses would not always be possible, especially in large-scale studies. Therefore, the combination of subjective rating and seat preference methods is suggested as appropriate methods for assessing daylight perception. Future research should also consider the impact of other environmental parameters on seat preference and how they relate to lighting conditions to improve occupant satisfaction. The interaction between any parameter and seating choice should not be examined in isolation; other aspects, such as privacy, outdoor view and quietness, should also be considered. Inter-individual differences in daylight perception are also worth investigating further.

## Data Availability Statement

The original contributions presented in the study are included in the article/supplementary material, and further inquiries can be directed to the corresponding author.

## Ethics Statement

The studies involving human participants were reviewed and approved by the UCL Research Ethics Committee. The patients/participants provided their written informed consent to participate in this study.

## Author Contributions

GI conducted the research and reported the results. HA (primary Ph.D. supervisor), JU (secondary Ph.D. supervisor), and CT provided feedback. All authors contributed to the article and approved the submitted version.

## Conflict of Interest

The authors declare that the research was conducted in the absence of any commercial or financial relationships that could be construed as a potential conflict of interest.

## Publisher’s Note

All claims expressed in this article are solely those of the authors and do not necessarily represent those of their affiliated organizations, or those of the publisher, the editors and the reviewers. Any product that may be evaluated in this article, or claim that may be made by its manufacturer, is not guaranteed or endorsed by the publisher.

## Appendix 1

**Table:** Details of the rooms and technical properties of the surfaces.

		Room 1	Room 2	Room 3
**Room geometry (m)**	Depth	6.50	19.30	15.20
	Width	4.70	10.30	7.00
	Height	2.81	3.75	2.79
**Room reflectance** ^*^	Floor (carpet)	0.03	0.03	0.03
	Walls	0.85 and 0.24	0.77	0.85
	Ceiling	0.79	0.79	0.79
	Window frame	0.81	0.81	–
**Furniture reflectance**	Desk	0.64	0.23	0.67
	Territory element	0.06 and 0.10	0.50	0.13
	Bookshelves	–	0.27	–
**Opening geometry (m)**	Number of openings	2 windows	13 windows	2 skylights
	Height x Width	1.99 × 1.25	2.58 × 1.25 and 2.58 × 1.68	–
	Width x Depth	–	–	3.20 × 6 and 3.20 × 1.80
**Glazing characteristics**	Visible transmission	0.60	0.60	0.60
**Blinds**		No	Yes—Occupancy controlled internal blinds	No
**Orientation**		N	N—E	–
**Outdoor view characteristics**		Church	Church and back building facade	Only sky view

* KONICA MINOLTA Illuminance meter T-10A (20014862) and KONICA MINOLTA Luminance gun meter LS 100 were used to measure surface illuminance and surface luminance, assuming perfectly diffusing surfaces, and this formula “Luminance = Reflectance x Illuminance/π” was applied to calculate reflectance of the surfaces.
